# The antibodies against the A137R protein drive antibody-dependent enhancement of African swine fever virus infection in porcine alveolar macrophages

**DOI:** 10.1080/22221751.2024.2377599

**Published:** 2024-07-08

**Authors:** Xiaoke Yang, Encheng Sun, Huanjie Zhai, Tao Wang, Shida Wang, Yuxuan Gao, Qinghe Hou, Xiangyu Guan, Shuwen Li, Lian-Feng Li, Hongxia Wu, Yuzi Luo, Su Li, Yuan Sun, Dongming Zhao, Yongfeng Li, Hua-Ji Qiu

**Affiliations:** a State Key Laboratory for Animal Disease Control and Prevention, National High Containment Facilities for Animal Diseases Control and Prevention, Harbin Veterinary Research Institute, CAAS, Harbin, People’s Republic of China; b Institute of Western Agriculture, CAAS, Changji, People’s Republic of China

**Keywords:** African swine fever virus, antibody-dependent enhancement, A137R protein, viral replication, Fc gamma receptors

## Abstract

African swine fever virus (ASFV) is the causative agent of African swine fever (ASF), a highly contagious disease that can kill up to 100% of domestic pigs and wild boars. It has been shown that the pigs inoculated with some ASF vaccine candidates display more severe clinical signs and die earlier than do pigs not immunized. We hypothesize that antibody-dependent enhancement (ADE) of ASFV infection may be caused by the presence of some unidentified antibodies. In this study, we found that the ASFV-encoded structural protein A137R (pA137R) can be recognized by the anti-ASFV positive sera, indicating that the anti-pA137R antibodies are induced in the ASFV-infected pigs. Interestingly, our results demonstrated that the anti-pA137R antibodies produced in rabbits or pigs enhanced viral replication of different ASFV strains in primary porcine alveolar macrophages (PAMs), the target cells of ASFV. Mechanistic investigations revealed that anti-pA137R antibodies were able to promote the attachment of ASFV to PAMs and two types of Fc gamma receptors (Fc*γ*Rs), Fc*γ*RII and Fc*γ*RIII, mediated the ADE of ASFV infection. Taken together, anti-pA137R antibodies are able to drive ASFV ADE in PAMs. These findings shed new light on the roles of anti-ASFV antibodies and have implications for the pathophysiology of the disease and the development of ASF vaccines.

## Importance

African swine fever (ASF) is a highly contagious disease that poses a risk to the world’s pig business. There is no licensed safe and effective vaccine except in Vietnam. One of the factors impeding the development of vaccines could be antibody-dependent enhancement (ADE). The causative agent of ASF is African swine fever virus (ASFV), which infects monocytes and macrophages and induces the production of atypical neutralizing antibodies, which is a common feature of viral infection that can induce ADE. In this study, the antibodies against the ASFV-encoded structural protein A137R (pA137R) are present in the convalescent sera from the ASFV-infected pigs and can promote viral replication of the CD2v-deleted ASFV mutant, genotype II wild-type ASFV, genotype I/II recombinant ASFV, but not the *A137R*-deleted ASFV mutant. Interestingly, the anti-pA137R antibodies are able to promote the attachment of ASFV to primary porcine alveolar macrophages. We conclude that anti-pA137R antibodies can drive the ASFV ADE mediated by two types of Fc gamma receptors (Fc*γ*Rs), Fc*γ*RII and Fc*γ*RIII. Therefore, ADE should be taken into account in the rational design of novel ASF vaccines.

## Introduction

The ability of antibodies to promote the clearance of the virus-infected cells *via* the Fc receptors-mediated immune responses holds significant potential for conferring exceptional immunological protection [1–3]. One of the mechanisms by which antibodies execute their functions involves eliciting antibody-dependent cell-mediated cytotoxicity (ADCC). For instance, antibodies facilitate ADCC to eliminate the cells infected with influenza virus [2,3]. However, despite their crucial roles in mediating immune protection, antibodies have the potential to promote the penetration of viruses into target cells and enhance viral infection, which is known as antibody-dependent enhancement (ADE) phenomenon [4]. The ADE observed in the viruses that infect monocytes and macrophages can be driven by cross-reactive antibodies, non-neutralizing antibodies (non-NAbs), or sub-NAbs [5,6]. Consequently, it is imperative to thoroughly evaluate the potential to induce ADE of emerging viruses, which poses a challenge to the development of vaccines and antibody drugs.

ADE is an alternative mechanism of virus infection in the immune cells that is driven by antibodies and Fc gamma receptors (Fc*γ*Rs). ADE is especially important for viruses to infect cells expressing Fc*γ*Rs, which can be triggered by cross-reactive antibodies, non-NAbs, or sub-NAbs against the structural proteins or their epitopes. Macrophages are considered to be the major contributor to ADE, as they express Fc*γ*Rs on the cell surfaces, including Fc*γ*RI (CD64), Fc*γ*RII (CD32), and Fc*γ*RIII (CD16) [7]. The expression level and affinity of CD16 on macrophages are the highest, followed by CD32 [8]. For example, Fc*γ*RII and Fc*γ*RIII mediate the modest ADE of SARS-CoV-2 infection [9] and Fc*γ*RII mediates the ADE of porcine reproductive and respiratory syndrome virus (PRRSV) infection [10].

African swine fever (ASF), caused by African swine fever virus (ASFV), is a transboundary epidemic that is prevalent in many countries [11]. Vietnam has officially approved the use of gene-deleted vaccines, but fully compliant vaccines that fulfil with safety and efficacy standards are still far away [12]. Unfortunately, it has been shown that inactivated ASF vaccines pose a risk of increasing the severity of the disease, and some subunit ASF vaccines have been found to accelerate the death of pigs [13–15]. NAbs are essential for blocking viral infections. Nonetheless, in the case of ASFV infection, the production of typical NAbs becomes compromised, thus rendering the antibody responses intricate and multifaceted. Anti-p54 and -p30 antibodies inhibit the penetration of ASFV [16]. However, these antibodies are not able to provide complete protection against the challenge of highly virulent ASFV strains [17–19]. ASFV infects macrophages and monocytes and causes the creation of non-NAbs, suggesting that ASFV may potentially induce ADE. There are possibilities that ADE and ASFV infection are related, which could significantly impede the development of an ASF vaccine [20].

ASFV has a big, intricate genome that encodes more than 165 proteins, half of which remain to be understood. The A137R protein (pA137R or p11.5) of ASFV is identified as a late-expressed structural protein [21]. pA137R is located in the cytoplasm and colocalized with p72 in the “viral factories” of the perinuclear cytoplasmic area [21]. Cryo-electron microscopy using single-particle reconstruction has unveiled that pA137R is assembled into a dodecahedron cage, functioning as a pivotal component in the icosahedral ASFV virion [22]. Importantly, ASFV can be effectively captured and targeted by anti-pA137R monoclonal antibodies (MAbs), indicating the specific binding of the antibodies to ASFV particles [23]. The deletion of the *A137R* gene in the ASFV Georgia 2010 strain leads to a decrease in pathogenicity and provides complete immune protection, indicating that *A137R* is a virulence-related gene [24]. Moreover, our group demonstrated that pA137R involved in the regulation of ASFV virulence by inhibiting the cyclic GMP-AMP synthase (cGAS)-STING-mediated interferon beta (IFN-*β*) signalling cascade through lysosomal degradation of the TANK-binding kinase 1 (TBK1) [18]. Collectively, pA137R is incorporated into the virion as a core component in the icosahedral ASFV particles and associated with viral pathogenicity.

In this study, we found firstly that the antibodies against novel immunogenic protein pA137R may drive higher ASFV replication by promoting the attachment of ASFV to PAMs and both Fc*γ*RII and Fc*γ*RIII mediate the ADE of ASFV infection, providing direct evidence for ASFV ADE.

## Materials and methods

### Cell, viruses, and antibodies

PAMs and HEK293 T cells were cultured in Roswell Park Memorial Institute 1640 (RPMI 1640) medium (catalog no. C11875500BT; Gibco) supplemented with 10% fetal bovine sera (FBS) (catalog no. 10091148; Gibco) and 2% penicillin-streptomycin (catalog no. 15140122; Gibco) in a 37°C incubator with 5% CO_2_. The genotype II ASFV HLJ/18 strain (wild-type ASFV, ASFV-WT) (GenBank no. MK333180.1), the genotype I/II recombinant ASFV JS/LG/21 (GenBank no. OQ504956), the *A137R*-deleted mutant ASFV-ΔA137R, the CD2v-deleted mutant ASFV-ΔCD2v-EGFP, and the HEK293T-adapted ASFV strain ASFV-P121 were propagated as described previously [25–28]. The ASFV-convalescent or -non-convalescent swine sera were collected from the pig farms, mouse polyclonal antibodies against pH171R, pD117L, pCP123L, pE120R, pF317L, and pA137R were generated in our laboratory, and rabbit polyclonal antibodies against pA137R and negative sera were described previously [25]. Porcine polyclonal antibodies against pA137R and irrelevant IgG were home-made and purified by Biodragon (AbBox). The anti-CD16/CD32, anti-CD32, or anti-CD16 antibodies were commercially available (catalog no. 88280S; Cell Signaling Technology; catalog nos. ARG22888, AGR22980; Arigo).

### Construction of the plasmids and purification of the ASFV proteins

The *A137R* and *CP204L* genes were cloned into the pCAGGS-Flag vector (Clontech) to create pFlag-pA137R and pFlag-p30, respectively. The recombinant proteins pH171R, pD117L, pCP123L, pH124R, pE120R, pF317L, pE146L, pE184L, pCP530R(aa1-200), pA137R, p30, Cap, and GST were produced and purified in our laboratory.

### Immunoelectron microscopy (IEM)

PAMs seeded on plates were infected with ASFV-WT at a multiplicity of infection (MOI) of 5 for 18 h. Subsequently, the cells were fixed with 4% paraformaldehyde and 1% glutaraldehyde in 0.1 M HEPES solution at pH 7.2 at 4°C for 2 h. The dehydration was performed in 50%, 70%, 90%, and 100% dimethylformamide (DMF), respectively, at 4°C for 15 min. The cells were embedded in LR white resin and then polymerized under UV irradiation at −20°C for 10 d. Subsequently, the fixed cell sections were prepared and immunolabelled as described previously [29].

### Evaluation of ADE

PAMs were incubated in 96-well plates in a 37°C incubator with 5% CO_2_ for 12 h. The sera were inactivated at 56°C for 30 min and centrifuged at 12,000 × *g* for 5 min. Anti-pA137R antibodies were 2- or 10-fold diluted from 1:50 or 1:10 and mixed with different ASFV strains of an MOI of 1 at 37°C for 90 min. The negative sera were processed using an identical procedure. At 48 h postinfection (hpi), the viral genome copies and the infectious virus titers, quantified as median tissue culture infective doses (TCID_50_) or median hemadsorption doses (HAD_50_), were expressed as fold changes relative to the control. The data represented three independent experiments.

### Virus binding assay

In order to explore whether the anti-pA137R antibodies of serial dilutions affect the attachment of ASFV-WT to PAMs, the following experimental procedures were conducted. Firstly, the sera were inactivated at 56°C for 30 min and centrifuged at 12,000 × *g* for 5 min. The serum samples were subsequently diluted at 1:100 or an initial ratio of 1:10 or 1:50, followed by sequential 2-fold dilutions (ranging from 1:10 to 1:640 or from 1:50 to 1:3200). In addition, the same treatment was performed with negative sera, as for the control group. The ASFV with an MOI of 1, 3, or 5 was incubated with the above samples at 37°C for 90 min at a ratio of 1:1. Finally, the virus-sera mixture was added to the PAMs cultured in 24-well plates, and incubated at 4°C for 2 h. The cells were washed with pre-cooled phosphate-buffered saline (PBS) to remove the unbounded virus and subjected to DNA extraction.

### Quantitative real-time PCR (qPCR)

ASFV genomic DNA was extracted from the cells using the MagaBio plus virus DNA purification kit (catalog no. 9109; BioFlux) according to the manufacturer’s protocols. ASFV genomic copies were quantified by qPCR on the QuantStudio system (Applied Biosystems, USA) as described previously [29].

### Hemadsorption assay

Since the CD2v protein is responsible for the hemadsorption of the ASFV-infected cells in the presence of porcine red blood cells, a hemadsorption assay was utilized to evaluate the TCID_50_ of ASFV [29,30]. Around 10^5^ PAMs seeded in 96-well plates were infected with ASFV for 48 h. Then, the cells were incubated with 10^6^ porcine red blood cells diluted in RPMI 1640, and hemadsorption was observed at 5 days postinfection (dpi).

### Indirect fluorescent assay (IFA)

HEK293 T in 24-well plates were infected with ASFV-P121 and incubated in an incubator with 5% CO_2_ at 37°C for 48 h. Each well was fixed with 0.2 mL of paraformaldehyde and permeabilized with 0.2 mL of 0.2% Triton X-100 (Sangon Biotech). Then, the cells were incubated with homemade rabbit or porcine anti-pA137R or negative sera (1:100) at 37°C for 2 h. After four washes with PBS containing 0.05% tween 20 (PBST) and rinsed with PBS, the cells were stained with fluorescein isothiocyanate (FITC)-labelled goat anti-rabbit/pig IgG (1:300) (Roche) for 50 min at 37°C. Finally, all the samples were washed with PBST four times and PBS twice and observed under an inverted fluorescence microscope (EVOS FL; Life Technologies).

### Indirect enzyme-linked immunosorbent assay (ELISA)

The ELISA plates coated with 100 μL of the purified recombinant pA137R (6.25 µg/mL) or GST (6.25 µg/mL) diluted in carbonate buffer (34 mM Na_2_CO_3_, 100 mM NaHCO_3_, pH 9.6) were incubated at 4°C overnight. After three washes with PBST, the plates were blocked at 37°C for 2 h using 180 μL of blocking buffer (Surmodics, USA). Subsequently, the ASFV-convalescent sera were diluted in PBS (1:100) and added to each well of the plates after removing the blocking buffer. Following incubation at 37°C for 1 h, the plates were washed three times with PBST, and horseradish peroxidase (HRP)-conjugated goat anti-pig IgG (diluted at 1:20,000 in the StabilZyme SELECT Stabilizer, Surmodics, USA) was added to each well at 37°C for 1 h. After three washes with PBST, 100 µL of the chromogenic substrate solution (TMB, Sigma-Aldrich, USA) was added. The color reaction was developed for 30 min and then stopped by adding 50 µL of 2 M sulfuric acid. The optical density (OD) values at 450 nm (OD_450nm_) of the reactions were measured using a microplate reader (Biotek, USA). The concentrations of antigens and antibodies were optimized via a checkerboard titration.

### Western blotting

The samples were mixed with 5 × SDS-PAGE loading buffer, boiled for 13 min, and centrifuged at 12,000 × *g* for 5 min. Following 12.5% SDS-PAGE electrophoresis, the gel was electro-transferred to a nitrocellulose membrane (Bio-Rad, USA). The convalescent sera from the pigs that recovered from ASFV infection or the non-convalescent sera from the ASFV-infected pigs (1:100 dilution) were used as primary antibodies to detect the immunogenicity of the target proteins. IRDve 800CW goat anti-pig IgG (1:10,000 dilution) was used as the secondary antibody. Finally, the bands were visualized using the Odyssey infrared imaging system (Li-Cor Biosciences).

### Antibody blocking assay

PAMs were incubated with anti-CD16/CD32, anti-CD32 or anti-CD16 antibodies of different concentrations at 37°C for 1 h. Porcine anti-pA137R antibodies (at 1:100 dilution) were incubated with ASFV-WT at 37°C for 90 min. Subsequently, the mixture (virus-antibodies) was then added to the PAMs and incubated for 24 h. The qPCR and viral titration assays were then performed as described above.

### Statistical analysis

SPSS 22.0 software (SPSS Software, Inc.) was used to analyze all data. The statistical significance of differences between groups was assessed using Student’s *t* test. An unadjusted *P*-value of less than 0.05 was considered to be significant.

## Results

### Anti-pA137R antibodies have the potential to enhance viral infection

**Figure 1.**
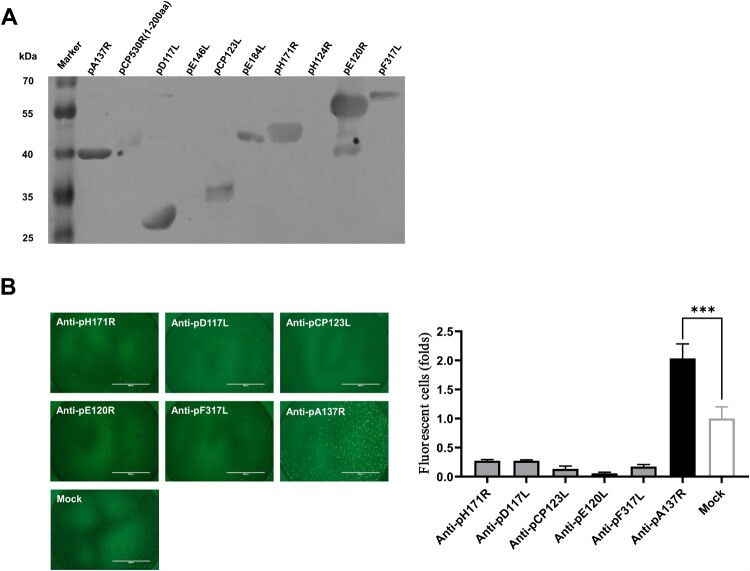
Anti-pA137R antibodies have the potential to enhance viral infection. **(A)** Identification of the new ASFV immunogenic proteins. The reactivity of ASFV structural proteins with the convalescent sera was analyzed by western blotting. **(B)** Mouse anti-pA137R sera augment ASFV infection. ASFV-ΔCD2v-EGFP was incubated with antibodies against the novel immunogenic proteins or the negative sera for 90 min. At 48 h postinfection, the fluorescent cells infected with ASFV-ΔCD2v-EGFP were observed under a fluorescent microscope.

To date, the immunogenicity of ASFV proteins remains largely unknown. Pigs that survive ASFV infection can serve as a novel tool for screening immunogenic proteins. The reaction of the ASFV structural proteins with ASFV-convalescent porcine sera was investigated by western blotting analysis. The results showed that seven of the structural proteins react with the convalescent sera, including pA137R, pD117L, pCP123L, pE184L, pH171R, pE120R, and pF317L, suggesting that the seven structural proteins of ASFV can induce antibody response and are identified as novel immunogenic proteins (Figure 1A).

Next, we prepared the mouse polyclonal antibodies against the six newly identified immunogenic proteins and examined the anti-ASFV activities by virus neutralization assay. Unexpectedly, the results showed that the fluorescent cells infected with ASFV-ΔCD2v-EGFP were significantly increased when it incubated with the anti-pA137R antibodies in contrast with the negative sera, while the antibodies against other five proteins inhibit ASFV replication (Figure 1B). Collectively, in contrast to other antibodies against the five proteins, anti-pA137R antibodies have the potential to enhance ASFV infection.

### Anti-pA137R antibodies are detectable in ASFV-convalescent porcine sera

**Figure 2.**
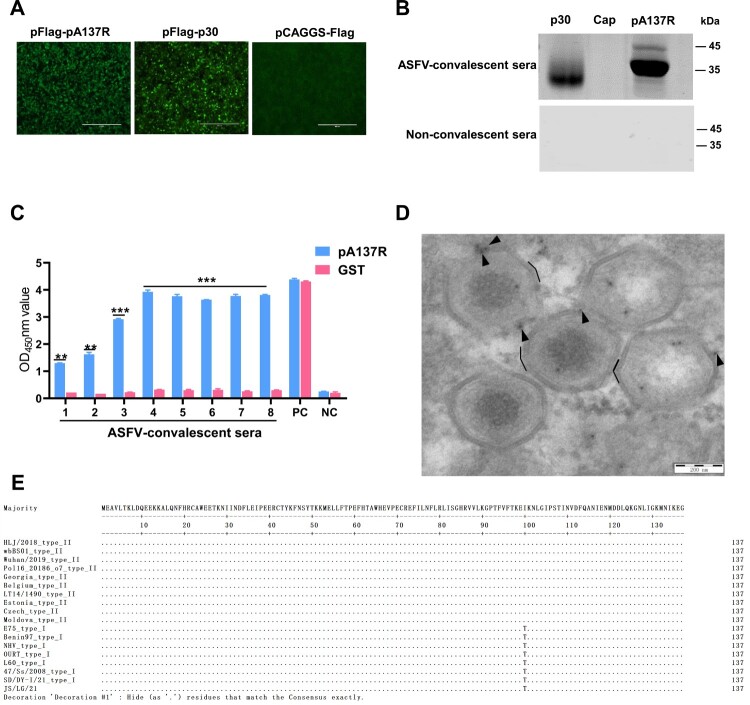
Anti-pA137R antibodies are detectable in the convalescent sera from ASFV-infected pigs. **(A)** The ASFV pA137R specifically reacts with the convalescent sera from the pigs that survived ASFV infection, while the non-convalescent sera from the ASFV-infected pigs were used as a negative control. HEK293 T cells were transfected with pFlag-A137R (2 μg), pFlag-p30 (2 μg), or pCAGGS-Flag (serving as a negative control) for 48 h and then the fluorescence was analyzed by IFA. **(B)** The reactivity of the purified pA137R with ASFV-convalescent or non-convalescent sera examined by western blotting. The p30 and Cap proteins were used as positive and negative controls, respectively. **(C)** Anti-pA137R antibodies are induced in ASFV-infected convalescent pigs. The antibodies against pA137R were tested in the sera collected from the ASFV-infected pigs by the indirect ELISA using the recombinant pA137R or GST as coated antigens. **(D)** Subviral localization of pA137R. ASFV-infected PAMs were fixed at 18 h postinfection and immunolabelled with a rabbit anti-pA137R antibodies followed by an anti-rabbit antibody conjugated to 5-nm-diameter gold particles. The arrowheads indicate the gold particles present on outer protein capsid of intracellular virus particles and the capsid is depicted in black line. Bar, 200 nm. **(E)** Multiple sequence alignment of pA137R of diverse ASFV isolates. A conservation analysis of the pA137R among genotypes I, II, and I/II recombinant ASFV isolates was conducted using the Jalview and ClustalW algorithms.

Since anti-pA137R antibodies have the potential to promote viral infection, whether antibodies against pA137R are present in the sera from the ASFV-infected pigs was investigated. To this end, the reactivity of pA137R with ASFV-convalescent porcine sera was assessed by western blotting and IFA, while the non-convalescent sera from the ASFV-infected pigs were used as a negative control. The results indicated that pA137R was highly immunogenic (Figure 2A and B). Anti-pA137R antibodies were detected in the sera collected from the ASFV-convalescent pigs by ELISA using pA137R, but not GST, as a coated antigen (Figure 2C), suggesting that ASFV infection elicited anti-pA137R antibodies and prompted us to determine whether it could elicit the ASFV ADE. As shown in Figure 2D, the specifically labelled pA137R exhibited an association with the periphery of intracellular virus particles and the radial dispersion of the gold particles aligned with the position of outer protein capsid of the intracellular virus particle. This observation suggests that anti-pA137R antibodies are capable of binding to ASFV virions. In addition, since the pA137R of the genotype I/II recombinant ASFV isolate JS/LG/21 originates from a genotype I ASFV strain (28), the amino acid conservation of pA137R among genotypes I, II, and I/II recombinant ASFV isolates was analyzed with the Jalview and ClustalW algorithm. The results revealed that only one amino acid site mutation was observed on the pA137R sequences of the ASFV isolates of the related genotypes (Figure 2E).

### Rabbit anti-pA137R antibodies induce ADE for ASFV in PAMs

**Figure 3.**
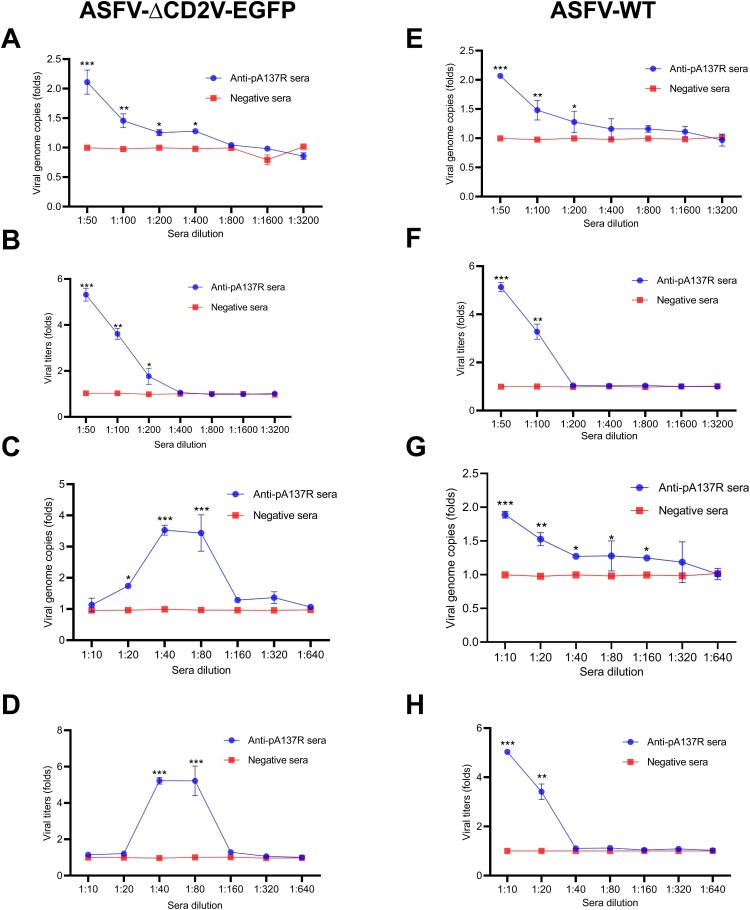
Rabbit anti-pA137R antibodies induce the ASFV ADE in PAMs. **(A and B)** Increased ASFV-ΔCD2v-EGFP replication by rabbit anti-pA137R antibodies of dilutions from 1:50 to 1:3200 in PAMs. PAMs were infected with ASFV-ΔCD2v-EGFP in the presence or absence of anti-pA137R antibodies. The viral genome copies were quantified by qPCR, and the viral titers (Log_10_ TCID_50_/mL) were titrated at 48 h postinfection (hpi). **(C and D)** Enhanced ASFV-ΔCD2v-EGFP replication by anti-pA137R antibodies at dilutions from 1:10 to 1:640 in PAMs. The viral genome copies, and viral titers (Log_10_ TCID_50_/mL) were determined following infection with the complex of ASFV-ΔCD2v-EGFP and the sera. **(E and F)** Enhanced ASFV-WT replication by anti-pA137R antibodies in PAMs. The replication of ASFV-WT in the presence of anti-pA137R antibodies with serial dilutions from 1:50 to 1:3200 was examined. The viral genome copies and viral titers (Log_10_ HAD_50_/mL) of ASFV-WT were determined at 48 hpi. **(G and H)** Anti-pA137R antibodies at high concentration enhanced ASFV-WT infection in PAMs. The experimental data of each group were separately recorded under the treatment of a higher concentration of anti-pA137R antibodies with serial dilutions from 1:10 to 1:640. The viral genome copies were quantified by qPCR, and the viral titers of ASFV-WT were titrated as Log_10_ HAD_50_/mL at 48 hpi. Specific-pathogen-free rabbit sera were used as a negative control. The data represented three independent experiments. All the data were expressed as fold changes relative to the control. The error bars represented the standard errors of the means. All the data were analyzed using the Student’s *t* test: ***, *P* < 0.001; **, *P* < 0.01; *, *P* < 0.05; ns, not significant, *P* > 0.05.

Based on the above results, whether the rabbit anti-pA137R antibodies promote viral replication was further evaluated. PAMs were infected with ASFV-ΔCD2v-EGFP in the presence or absence of the serially diluted rabbit anti-pA137R antibodies (starting at 1:50). The results showed that the anti-pA137R antibodies of lower than 1:200 dilution promoted ASFV-ΔCD2v-EGFP infection by quantification analysis of the genome copies by qPCR (Figure 3A) and viral titers by titration (Figure 3B). The effects of the sera at lower dilutions (beginning at 1:10) on the viral replication were further investigated. The results demonstrated that the sera diluted at 1:40 and 1:80 exhibited the ability to augment viral replication in PAMs. As expected, the negative sera did not affect the replication of ASFV in PAMs (*P* > 0.05) (Figure 3C and D). These data indicate that anti-pA137R antibodies at a certain dilution potently enhance ASFV-ΔCD2v-EGFP infection in PAMs.

Next, the enhancement of ASFV-WT infection by the rabbit anti-pA137R antibodies was examined. The results showed that anti-pA137R antibodies diluted at 1:50 led to enhanced ASFV-WT infection (Figure 3E and F). Furthermore, the effect of lower dilution serum on the replication of ASFV-WT was also determined. The results showed that the infection of ASFV-WT was significantly increased by anti-pA137R antibodies diluted at 1:10 (Figure 3G and H). Remarkably, the data showed that the infection of ASFV-WT was significantly increased.

Collectively, these data demonstrate that anti-pA137R antibodies produced in rabbits enhance the infection of ASFV-ΔCD2v-EGFP or ASFV-WT in PAMs.

### Porcine anti-pA137R antibodies drive the ADE of ASFV-WT but not the A137R-deleted ASFV mutant in PAMs

**Figure 4.**
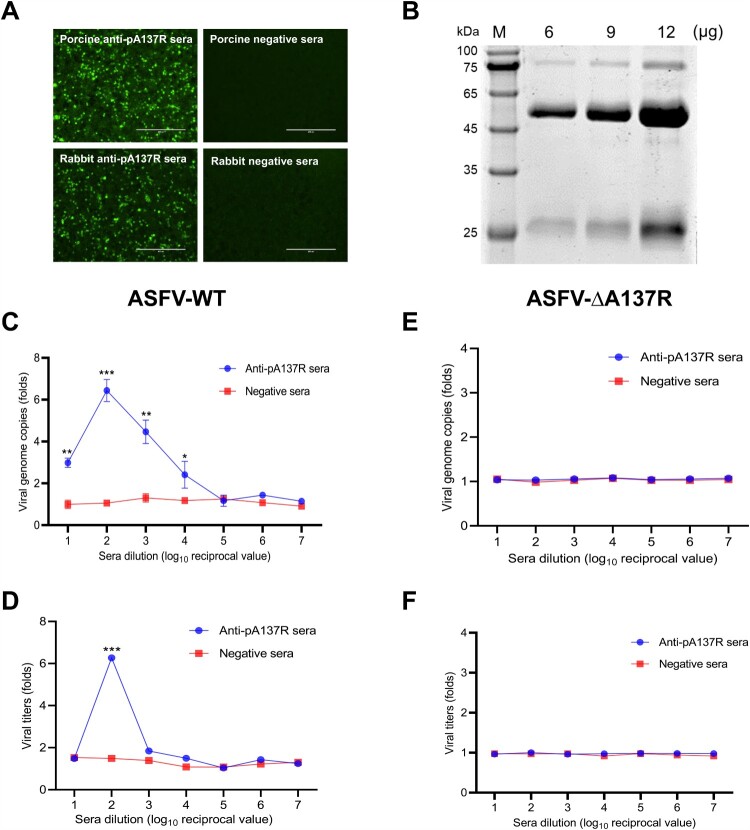
Porcine anti-pA137R antibodies drive ADE of ASFV-WT but not of ASFV-ΔA137R in PAMs. **(A and B)** The reactivity and purity of the purified anti-pA137R antibodies. **(C and D)** Porcine anti-pA137R antibodies increase ASFV-WT replication in PAMs. Anti-pA137R antibodies and negative sera at 10-fold serial dilutions starting 1:10 were incubated with ASFV-WT, and the mix were added to PAMs to determine the enhancement folds of the viral genome copies and titers. **(E and F)** Anti-pA137R antibodies do not affect the replication of ASFV-ΔA137R in PAMs. After a 1.5-h incubation of anti-pA137R antibodies or negative sera of serial dilutions with ASFV-ΔA137R, the viral genome copies and titers of each group were determined. Specific-pathogen-free porcine sera were used as a negative control. The data represented three independent experiments. The error bars represent the standard errors of the means. All the data were analyzed using the Student’s *t* test: ***, *P* < 0.001; **, *P* < 0.01; *, *P* < 0.05; ns, not significant, *P* > 0.05.

To further figure out whether the ASFV ADE can be induced by porcine anti-pA137R antibodies, the porcine polyclonal antibodies against pA137R were prepared and purified (Figure 4A and B). ASFV-WT was preincubated with a serially diluted porcine anti-pA137R antibodies (starting at 1:10) and then used to infect PAMs. The results showed that the negative sera did not affect ASFV infection, while the infection of ASFV-WT was enhanced significantly by the sera at a dilution of 1:100, showing a typical ADE curve (Figure 4C and D).

Next, we tested the effects of anti-pA137R antibodies of serial dilutions on the replication of the *A137R*-deleted mutant ASFV-ΔA137R in PAMs. As expected, the anti-pA137R antibodies did not affect replication of the mutant ASFV-ΔA137R in PAMs, as did the negative sera (Figure 4E and F).

### Porcine anti-pA137R antibodies drive ADE of the genotype I/II recombinant ASFV strain

**Figure 5.**
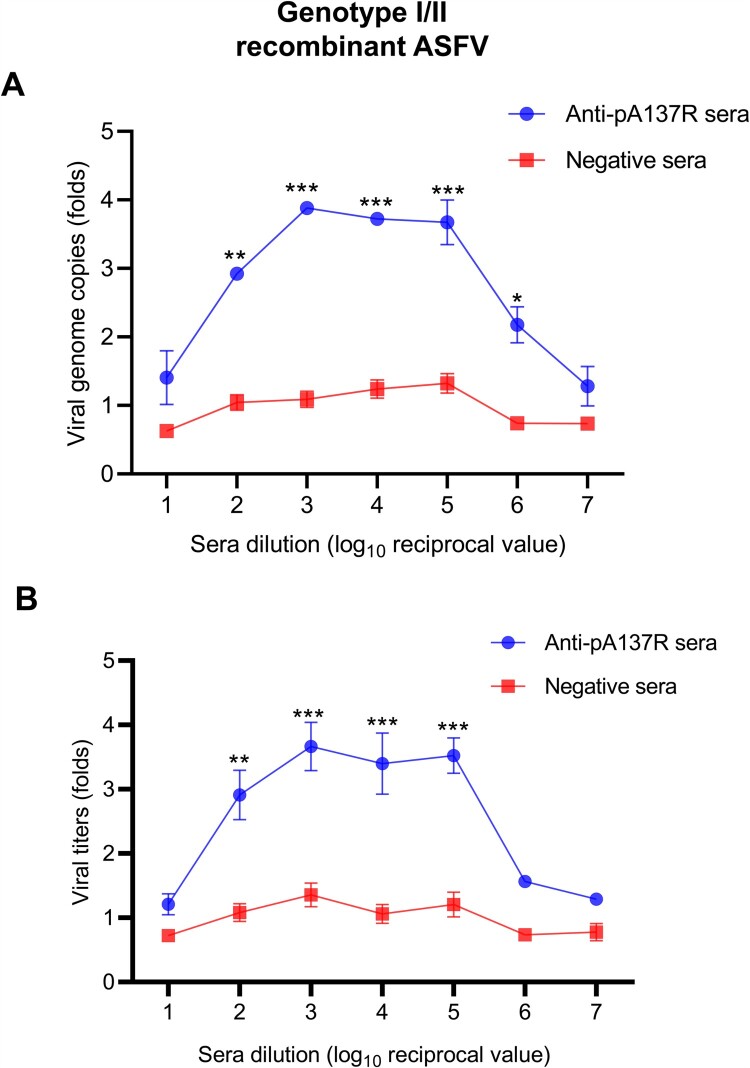
Porcine anti-pA137R antibodies drive ADE of the genotype I/II recombinant ASFV strain. **(A and B)** The genotype I/II recombinant ASFV strain was incubated with anti-pA137R antibodies of different concentrations. Specific-pathogen-free pig sera were used as a negative control. The mixture was added to PAMs to determine the enhancement folds of the viral genome copies and titers. The data represented three independent experiments. The error bars represent the standard errors of the means. All the data were analyzed using the Student’s *t* test: ***, *P* < 0.001; **, *P* < 0.01; *, *P* < 0.05; ns, no significance.

The ADE of dengue virus (DENV) infection occurs in infections with different genotypes [31]. Based on the C-terminal sequence of the ASFV *B646L* gene, ASFV includes 24 genotypes with 86.2% to 99.5% nucleotide identities [32]. The pA137R-encoding sequence was highly conserved, and there was only one amino acid mutation on the pA137R of the genotypes I and II ASFV strains. To better understand the cross-reactivity of anti-pA137R antibodies against different genotypes of ASFV, we evaluated the infectivity of the genotype I/II recombinant ASFV strain that was incubated with anti-pA137R antibodies of serial dilutions. Although anti-pA137R antibodies concentration that most effectively enhanced infection with the recombinant ASFV strain was different from ASFV-WT, an ADE curve of the genotype I/II recombinant ASFV was also observed (Figure 5A and B). In summary, these results demonstrate that anti-pA137R antibodies produced in pigs drive ADE of the recombinant ASFV strain in PAMs.

### Anti-pA137R antibodies facilitate the attachment of ASFV-WT to PAMs

**Figure 6.**
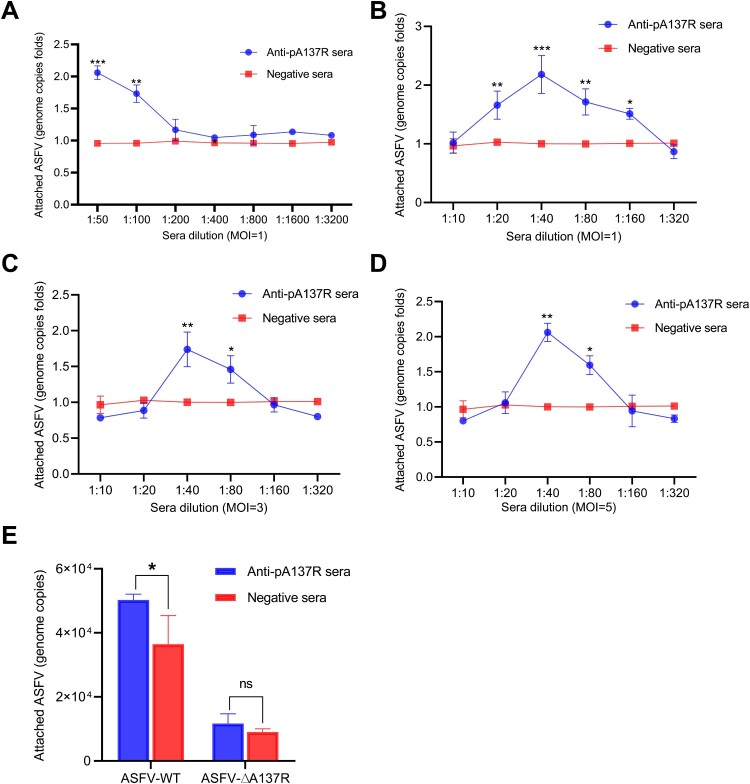
Porcine anti-pA137R antibodies promote the attachment of ASFV-WT to PAMs. **(A**–**D)** Rabbit anti-pA137R antibodies facilitate the attachment of ASFV-WT to PAMs. To check the effects of anti-pA137R antibodies of serial dilutions on the attachment of ASFV to the target cells, PAMs were infected with ASFV-WT at an MOI of 1, 3, or 5 in the presence or absence of anti-pA137R antibodies of serial dilutions. After a 2-h incubation at 4°C, the ASFV genomic DNA was extracted and quantified by qPCR (the results were shown as fold changes). **(E)** Porcine anti-pA137R antibodies promoted the attachment of ASFV-WT but not ASFV-ΔA137R to PAMs. The viral genome copies of ASFV-WT and ASFV-ΔA137R in the presence of porcine anti-pA137R antibodies or negative sera of 1:100 dilution were quantified by qPCR. The data represented three independent experiments. The error bars represented the standard errors of the means. All the data were analyzed using the Student’s *t* test: ***, *P* < 0.001; **, *P* < 0.01; *, *P* < 0.05; ns, not significant, *P* > 0.05.

Viral infection is a multistep process, including the early entry and replication stage and the late assembly and release stage [33]. Notably, ADE is involved in the entry of various viruses into the cell [34–36]. To investigate how anti-pA137R antibodies produced in rabbits affected the early infection, the binding ability of ASFV to cells was analyzed by qPCR. Negative sera did not affect the attachment of ASFV to PAMs, whereas anti-pA137R antibodies could promote ASFV-WT attachment below 1:200 dilution (Figure 6A and B).

In order to further verify whether the effects of anti-pA137R antibodies on the attachment of ASFV-WT to PAMs depend on the infection dose, the attachment of the virus to PAMs at higher MOIs was further examined. Consistent results were observed at an MOI of 3 or 5 (Figure 6C and D).

Furthermore, the different attachment abilities of ASFV-WT and ASFV-ΔA137R incubated with porcine anti-pA137R antibodies at 1:100 (the dilution showing a peak enhancement) were tested. Anti-pA137R antibodies promoted the attachment of ASFV-WT, but did not affect the attachment of ASFV-ΔA137R (Figure 6E). Collectively, these data suggest that anti-pA137R antibodies produced in rabbits or pigs at a certain concentration promote the attachment of ASFV-WT but not ASFV-ΔA137R to PAMs.

### Fc*γ*RII and Fc*γ*RIII are involved in the ASFV ADE

**Figure 7.**
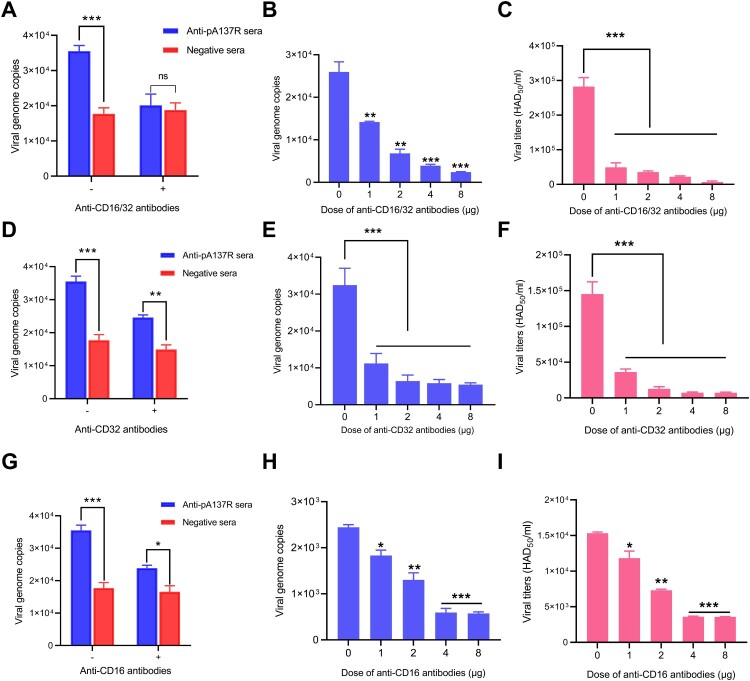
Fc*γ*RII and Fc*γ*RIII are involved in the ASFV ADE. **(A–C)** Inhibition of ASFV ADE in the presence of anti-CD16/CD32 antibodies in a dose-dependent manner. Using different amounts of anti-CD16/CD32 antibodies to block the Fc*γ*RII and Fc*γ*RIII on the surface of PAMs, subsequently the PAMs were infected using the complex of ASFV-WT and anti-pA137R antibodies and incubated for 24 h. **(D–I)** ADE of ASFV infection is mainly mediated by Fc*γ*RII or Fc*γ*RIII. Different amounts of anti-CD16 or anti-CD32 antibodies were inoculated with PAMs. ASFV-WT in the presence of porcine anti-pA137R antibodies or negative sera of 1:100 dilution was added to the PAMs. At 24 h postinfection, the viral genome copies were quantified by qPCR, and the viral titers of ASFV-WT were titrated as Log_10_ HAD_50_/mL. The data represented three independent experiments. The error bars represented the standard errors of the means. All the data were analyzed using the Student’s *t* test: ***, *P* < 0.001; **, *P* < 0.01; *, *P* < 0.05; ns, not significant, *P* > 0.05.

Since ADE is mediated by the Fc*γ*Rs on the cell surface, we conducted an antibody blocking assay to investigate which Fc*γ*R(s) is involved in the ASFV ADE. Fc*γ*RI, Fc*γ*RII, and Fc*γ*RIII are all expressed on the surface of PAMs. Considering the low-level expression of Fc*γ*RI, we employed anti-Fc*γ*RII and anti-Fc*γ*RIII antibodies of different concentrations. The results revealed that the mixture of anti-Fc*γ*RII and anti-Fc*γ*RIII antibodies effectively suppressed the ASFV ADE driven by anti-pA137R antibodies in a dose-dependent manner (Figure 7A to C).

Next, the anti-Fc*γ*RII or anti-Fc*γ*RIII antibodies of various amounts were employed to reduce the ADE of ASFV infection, yielding consistent results. This suggests that both Fc*γ*RII and Fc*γ*RIII mediate the ADE of ASFV infection (Figure 7D to I).

## Discussion

The development of several vaccines has been severely hampered by the existence of ADE. There isn’t a safe and efficient ASF vaccine on the market right now. ADE may be involved in ASFV infection, which significantly hinders the vaccine development. ASFV encodes more than 165 proteins, including 68 structural proteins [37]. However, the ADE-associated ASFV proteins are completely unknown. It is intriguing to note that anti-pA137R antibodies enhanced ASFV replication in PAMs.

pA137R is a most abundant viral protein that is highly expressed during ASFV replication cycle [38–40]. Furthermore, an ELISA has been developed to detect anti-pA137R antibodies [41]. These reports indicate that pA137R is a strong immunogen. In this study, convalescent sera from the ASFV-infected pigs further demonstrated the antigenicity of pA137R. Significantly, six ASFV strains were detected in a double-antibody sandwich ELISA using an anti-pA137R MAb [23]. Our results demonstrated for the first time that anti-pA137R antibodies are capable of binding to the intracellular ASFV virion, which are infectious as extracellular ASFV particles. The results are consistent with the report that pA137R is incorporated in the virion as a core component in the icosahedral ASFV particles [22]. Generally, the antibodies against the viral envelope or structural proteins can elicit ADE. For example, it has been indicated that the antibodies against the E or prM protein induce the DENV ADE [42]. In this study, pA137R was identified for the first time as the ADE-associated ASFV protein.

The sub- or non-NAbs may increase viral infection in the target cells [43]. Our data have confirmed that antibodies against multiple ASFV proteins and the sera of the ASF-recovered pigs can partially inhibit but not complete neutralize ASFV infection, indicating that ASFV infection does not elicit the typical NAbs. It has been shown that sub-NAbs against one serotype of a virus may lead to the ADE of another serotype of the virus, such as DENV [31]. The convalescent plasma collected from patients infected with early SARS-CoV-2 strains is able to induce ADE of SARS-CoV-2 variant infection [9]. We conducted the experiments to investigate the potential roles of anti-pA137R antibodies in promoting the ADE of the genotype I/II recombinant ASFV strain. Our findings reveal that anti-pA137R antibodies can induce ADE of the recombinant virus as well. Further research is required to determine whether the sera elicit the ADE of various genotypes of ASFV.

ADE has been found to play a role in the entry of numerous viruses, such as DENV, PRRSV, and coronaviruses [34–36,43,44]. In this work, we discovered that, at certain doses, anti-pA137R antibodies can facilitate ASFV attachment to PAMs. Increasing evidence has demonstrated that Fc*γ*Rs are involved in the ADE of coronaviruses, filoviruses, and flaviviruses [9,45,46]. Monocytes and macrophages, the target cells for ASFV infection, express Fc*γ*Rs on the cell surface. In this study, we showed that Fc*γ*RII and Fc*γ*RIII mediated the ADE of ASFV infection.

Furthermore, the viral infection in ADE circumstances modulates the host innate immune responses and alters the transcriptional levels of host molecules to facilitate viral replication [31]. For instance, DENV infection under ADE not only promotes the virus to enter the target cell but also inhibits the generation of type I IFNs, thus enhancing the replication in the host cells. It has been shown that both non-NAbs and highly diluted NAbs increase the replication of feline infectious peritonitis virus in macrophages and enhance the production of inflammatory cytokines, such as interleukin (IL)-1*β* and IL-6 [47]. As far as we currently know, the ASFV Georgia 2010 strain exhibits reduced pathogenicity and immunological protection as a result of the *A137R* gene deletion, but the molecular mechanisms are unclear [24]. Moreover, it has been demonstrated that pA137R negatively regulates the cGAS-STING-mediated IFN-*β* signalling pathway [25]. In this study, our findings further indicate that anti-pA137R antibodies increase ASFV replication in PAMs, while the enhancement is abolished with the *A137R* gene deleted. Therefore, as an ADE-related protein of ASFV, pA137R may also be involved in altering the host cell’s innate immune response. With the exception of how ADE affects viral entry, the molecular mechanisms for ASFV ADE involved in immune responses and transcription modulation remain unknown and are worthy of being investigated.

It has been shown that the clinical signs of the pigs immunized with some ASF subunit vaccines and live virus-vectored vaccines are more severe and death occurs earlier than those of the non-immunized pigs following challenge with virulent ASFV [14,48]. Immunization with inactivated ASF vaccines can enhance the severity of the disease [13]. Accordingly, ADE may play an important role in the pathogenesis of ASFV. Additionally, we also found that ASFV infection elicited anti-pA137R antibodies in pigs, which may increase the risk of ADE of ASFV infection in the field. The engagement of pA137R in the pathogenicity of ASFV in pigs will be investigated in the future.

Antibodies are not only unable to neutralize the virus but actively promote its infection, posing a serious obstacle to the creation of safe and efficacious vaccines. Numerous strategies are extensively implemented to mitigate the detrimental effects caused by the ADE of various viruses. For example, based on the investigation of the ADE mechanism of coronaviruses, subunit vaccines were designed devoid of the receptor binding domain of the spike protein to regulate viral infection [34,43]. In this study, we proved that anti-pA137R antibodies enhanced the replication of ASFV in PAMs. Identifying the viral proteins and epitopes responsible for ADE gives a significant clue to the development of safer vaccines. Therefore, we propose that pA137R should be excluded for the development of a safer and more effective ASF vaccines.

Conclusively, the antibodies targeting pA137R produced in rabbits or pigs can augment ASFV proliferation and facilitate viral attachment to PAMs, which offers fresh perspectives on the roles played by anti-ASFV antibodies and informs the development of future ASF vaccines.
